# Median Arcuate Ligament Syndrome

**DOI:** 10.7759/cureus.22106

**Published:** 2022-02-10

**Authors:** Taylor P Iobst, Kelsey M Lamb, Stacy L Spitzer, Ravi N Patel, Sameer S Alrefai

**Affiliations:** 1 General Surgery, Sentara Halifax Regional Hospital, Halifax, USA; 2 General Surgery, Edward Via College of Osteopathic Medicine, Blacksburg, USA

**Keywords:** laparoscopy, nonspecific abdominal pain, celiac axis, median arcuate ligament release, median arcuate ligament syndrome

## Abstract

Median arcuate ligament syndrome (MALS) is uncommon and often difficult to diagnose due to the vague presenting symptoms of abdominal pain, weight loss, and early satiety. Here, we report the case of a 63-year-old man who was successfully treated with laparoscopic median arcuate ligament release. Computed tomography (CT) of the abdomen and pelvis performed preoperatively demonstrated compression of the celiac artery with post-stenotic dilatation consistent with MALS. Subsequently, laparoscopic median arcuate ligament release was performed without any complications. Postoperatively, the patient reported resolution of abdominal pain with increased appetite and weight gain. Nonspecific abdominal pain and weight loss may raise concern for malignancy, but MALS should also be considered in the differential diagnoses. Diagnosis can be confirmed with CT and/or angiography. Median arcuate ligament release results in partial if not complete resolution of symptoms due to decompression of the celiac artery as well as division of the overlying celiac plexus.

## Introduction

Median arcuate ligament syndrome (MALS) is a rare diagnosis, found in 2 out of 100,000 patients [1]. MALS occurs when a thick band of tissue fibers, called the median arcuate ligament, presses on the celiac artery. The median arcuate ligament connects the right and left diaphragmatic crura on either side of the aortic hiatus proximal to the celiac trunk. In MALS, these fibers insert inferiorly and cross anteriorly to the celiac trunk causing compression of the artery [1]. This anomaly also causes compression of the overlying celiac plexus, which lies anteriorly to the aorta and is composed of afferent fibers from the upper abdominal viscera as well as sympathetic fibers from the greater and lesser splanchnic nerves.

Symptoms of MALS are nonspecific but can include chronic abdominal pain, postprandial epigastric pain, loss of appetite, nausea, vomiting, and weight loss [2]. Physical examination may demonstrate an epigastric abdominal bruit. Furthermore, compression of the celiac trunk is exacerbated with inspiration as the diaphragm moves inferiorly, and is alleviated with expiration [3]. Patients typically undergo an extensive evaluation including endoscopy and imaging before a diagnosis of MALS is made. Abdominal computed tomography (CT) demonstrates indentation of the proximal celiac trunk with a hooked appearance, which differentiates MALS from celiac artery atherosclerosis. The gold standard for diagnosis of MALS is three-dimensional computed tomography angiography (CTA), which can determine the degree of compression and identify potential collateral vessels from the superior mesenteric artery [3]. Other associated findings include post-stenotic dilatation and median arcuate ligament thickness (>4 mm) [4]. Magnetic resonance angiogram (MRA) and ultrasound techniques may also be used in diagnosis.

In our case, we discuss a case of MALS treated successfully with laparoscopic median arcuate ligament release and transection of the celiac plexus.

## Case presentation

A 63-year-old male with a medical history of chronic obstructive pulmonary disease, tobacco dependence, chronic alcohol use, and hypertension presented to his primary care physician for chronic abdominal pain, appetite loss, and a steady decline in weight. The patient reported eating only one meal per day and early satiety. He also reported several episodes of vomiting secondary to his severe abdominal pain. He denied any change in bowel or bladder habits. The patient had an extensive history of smoking and was being worked up for a solitary pulmonary nodule by pulmonology. 

**Figure 1:**
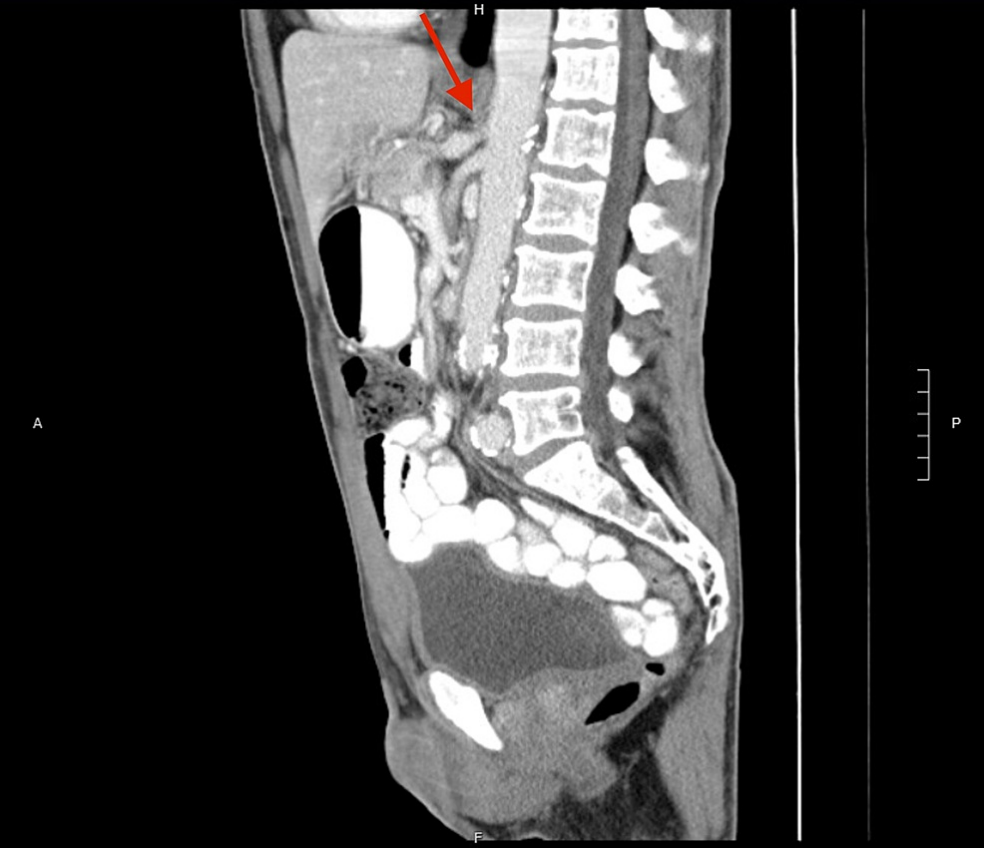
Preoperative CT of the abdomen/pelvis demonstrating celiac artery stenosis with post-stenotic dilatation consistent with MALS. CT: computed tomography; MALS: median arcuate ligament syndrome

Contrast-enhanced CT of the abdomen and pelvis revealed post-stenotic dilatation of the celiac axis suggestive of MALS (Figure 1).

The patient was subsequently referred to General Surgery for further evaluation and management. He continued to complain of abdominal pain, gastroesophageal reflux, nausea, and vomiting. Physical examination revealed an ill-appearing, cachectic male with abdominal tenderness. The patient also reported chronic constipation. His medical records did not reveal a previous colonoscopy.

He was referred to Gastroenterology for a colonoscopy and upper endoscopy. Upper endoscopy revealed *Helicobacter pylori* gastritis, which was subsequently treated with amoxicillin-clavulanate, clarithromycin, and omeprazole. His nausea and vomiting had resolved by his follow-up gastroenterology appointment; however, his abdominal pain and decreased appetite persisted. Due to inadequate symptom control and otherwise unremarkable workup, median arcuate ligament release was scheduled.

Abdominal access was obtained using a Veress needle inserted at Palmar’s point in the left upper quadrant. After insufflating the abdomen to 15 mmHg, a 5 mm Optiview trocar was inserted superior and left of the umbilicus. Subsequently, 5 Miller trochars were inserted in the left and right upper quadrants, and another 11 Miller trochar was inserted superior and to the right of the umbilicus. A small Nathanson retractor was used to retract the left lobe of the liver. First, the passive flaccida between the gastrohepatic ligament was opened to rule out a replaced left hepatic artery. The left gastric artery was identified, and blunt dissection was used to create a window around the left gastric artery. A Penrose drain was then placed around the left gastric artery to retract it laterally. The hepatic artery was identified and dissected from the adventitia using blunt dissection electrocautery. The right crus of the diaphragm was then identified, and the phrenoesophageal membrane was partially opened. The fibers of the right and left crura of the diaphragm were transected close to the aorta using blunt dissection and electrocautery. The aorta was identified, and the overlying fibrous band and muscle fibers were completely dissected using electrocautery. There was a firm compression from the abdominal aorta, which was preserved and dissected using blunt dissection. Oozing from a small branch of the abdominal aorta was controlled using a 5 mm clip applier. After identifying the compressed celiac trunk, transection of the median arcuate fibers was performed using the LigaSure device and blunt dissection with electrocautery. There was a small celiac plexus neurovascular bundle, which was transected using electrocautery. At the conclusion of dissection, the celiac trunk was free with no compression. After hemostasis was secure, the Nathanson retractor was removed, and the 11 mm trocar site was closed using the 0 PDS Endo Close device. The skin and subcutaneous tissue were infiltrated with 20 cc 1% lidocaine/0.25% marcaine. The skin was approximated using 4-0 Monocryl. Interrupted Dermabond was applied. The patient tolerated the procedure well.

**Figure 2:**
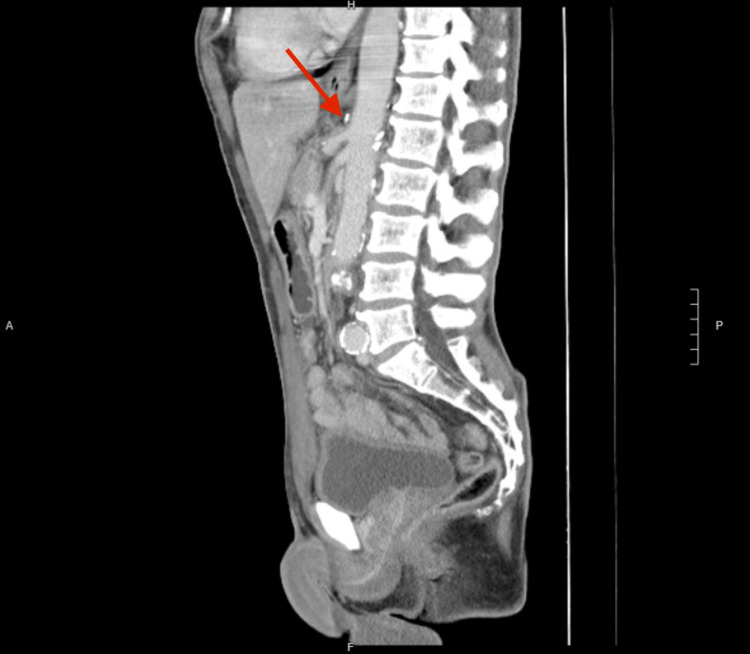
Postoperative CT scan of the abdomen/pelvis showing no evidence of celiac artery compression. CT: computed tomography

One month following the procedure, the patient reported feeling much better. He had gained weight, his appetite had improved, and his pain had resolved. Postoperative CT (Figure 2) showed no evidence of celiac artery compression.

## Discussion

MALS is a rare condition and usually a diagnosis of exclusion; however, a high level of clinical suspicion should remain in patients who present with unexplained abdominal pain and weight loss. Surgical intervention remains the definitive treatment option and involves laparoscopic ligament release.

Untreated MALS may have several potential sequelae, most notably the development of arterial aneurysms in the celiac artery and/or through collateral vessels associated with the superior mesenteric artery (SMA). Collateral circulation and retrograde blood flow via the SMA may develop in patients with long-standing MALS because of prolonged ischemia in the foregut organs supplied by the stenosed celiac artery [5]. With increased blood flow through these collateral vessels, there is increased risk of aneurysm formation and subsequent rupture. The development of collateral circulation is more likely to occur in peripancreatic vessels stemming from the SMA, including pancreaticoduodenal arcades and the dorsal pancreatic artery [6]. Once aneurysm formation through collaterals develops and has been detected through imaging, prophylactic embolization of the vessels, via coiling or stent placement, should be considered to reduce the risk of aneurysm rupture [5]. Splanchnic artery collateral formation may also explain the percentage of MALS cases that are asymptomatic and detected incidentally. Retrograde blood flow through the foregut can limit the postprandial abdominal pain and early satiety commonly experienced in MALS [7]. Collateral circulation and aneurysm formation should be considered in patients with MALS and may be prevented by early surgical intervention once a diagnosis has been made.

Treatment of MALS is surgical decompression with or without celiac plexus block. This was traditionally performed through an open approach; however, laparoscopic and robot-assisted approaches are increasing in frequency. Minimally invasive techniques allow for less postoperative pain and faster recovery. The major risk, regardless of approach, is injury to the aorta or its major branches with the potential need to convert to open surgery. The goal of surgery is to divide and excise the fibers of the median arcuate ligament that stretch across the celiac trunk. Intraoperative ultrasound can confirm reduced peak flow velocity through the celiac artery after decompression. The celiac plexus nerve fibers may also be divided during the procedure to aid in pain relief [8]. In a retrospective cohort study of 81 patients with clinically diagnosed MALS, 86% reported symptom relief after percutaneous celiac plexus block, with only 22% having CTA-confirmed celiac artery compression [9]. These results suggest that dissection of the celiac plexus at the time of median arcuate ligament release improves postoperative outcomes compared to celiac artery decompression alone.

If symptoms of nausea, vomiting, postprandial pain, and weight loss persist even after laparoscopic ligament release, revascularization of the celiac artery by either endovascular stenting or bypass can be considered as secondary options [2].

## Conclusions

We presented a case of MALS and the vague nature of its presentation. This case presentation highlights the importance of considering MALS in the differential diagnosis for patients presenting with chronic abdominal pain and symptoms worrisome for malignancy, including weight loss and early satiety.
